# Social representations of different generations about everyday life of homosexuals in modern Russia

**DOI:** 10.1192/j.eurpsy.2021.2158

**Published:** 2021-08-13

**Authors:** V. Lianguzova, A. Rikel

**Affiliations:** 1 Faculty Of Psychology, Moscow State University, Moscow, Russian Federation; 2 Psychology, Lomonosov Moscow State University, Moscow, Russian Federation

**Keywords:** Social representations, homosexuals, everyday life, Generations

## Abstract

**Introduction:**

The problem of homosexuality is constantly in the spotlight of the mass media, social media and politicians. At the same time, the cultural and national specificity of attitudes towards the phenomenon of homosexuality seems obvious, as well as a significant polarization of opinions within Russian society itself. With significant attention to this issue, there are not many attempts to analyze the socio-psychological basis of representations about homosexuality. At the same time, in a number of foreign studies it was revealed that the modern Z Gen is distinguished by greater tolerance and freedom of views in terms of attitude towards traditionally segregated social groups.

**Objectives:**

The purpose of this study was to identify representations about homosexuality among different generations of modern Russians.

**Methods:**

The methodological basis of the research was the study of the structure of social representations (Vergesse methodique). The research methods implied the author’s questionnaire aimed at identifying representations about homosexuality and a modified version of the RAHI questionnaire. The sample was N = 444 (residents of Russia, age 16-65).

**Results:**

There was shown a significant difference between the Z Gen in terms of tolerance of representations about homosexuality. So called ‘double standards’ were identified in terms of attitudes towards male and female homosexuality. The rooted concept of homosexuality as a relationship based, rather, on a sexual rather than a romantic-spiritual level of relationships, was stated.

**Conclusions:**

Main hypothesis was confirmed: an inverse relationship between age and perceptions of homosexuality as normative was revealed.

**Disclosure:**

No significant relationships.

